# Platinum Cyclooctadiene Complexes with Activity against Gram‐positive Bacteria

**DOI:** 10.1002/cmdc.202100157

**Published:** 2021-07-08

**Authors:** Angelo Frei, Soumya Ramu, Gabrielle J. Lowe, Hue Dinh, Lucie Semenec, Alysha G. Elliott, Johannes Zuegg, Anke Deckers, Nicole Jung, Stefan Bräse, Amy K. Cain, Mark A. T. Blaskovich

**Affiliations:** ^1^ Centre for Superbug Solutions Institute for Molecular Bioscience The University of Queensland St. Lucia QLD 4072 Australia; ^2^ ARC Centre of Excellence in Synthetic Biology Department of Molecular Sciences Macquarie University Sydney NSW Australia; ^3^ Institute of Organic Chemistry Karlsruhe Institute of Technology (KIT) Fritz-Haber-Weg 6 76131 Karlsruhe Germany; ^4^ Institute of Biological and Chemical Systems – Functional Molecular Systems (IBCS-FMS) Karlsruhe Institute of Technology (KIT) Hermann-von-Helmholtz-Platz 1 76344 Eggenstein-Leopoldshafen Germany

**Keywords:** antibiotic, metals in medicine, metalloantibiotic, inorganic medicinal chemistry, platinum

## Abstract

Antimicrobial resistance is a looming health crisis, and it is becoming increasingly clear that organic chemistry alone is not sufficient to continue to provide the world with novel and effective antibiotics. Recently there has been an increased number of reports describing promising antimicrobial properties of metal‐containing compounds. Platinum complexes are well known in the field of inorganic medicinal chemistry for their tremendous success as anticancer agents. Here we report on the promising antibacterial properties of platinum cyclooctadiene (COD) complexes. Amongst the 15 compounds studied, the simplest compounds Pt(COD)X_2_ (X=Cl, I, **Pt1** and **Pt2**) showed excellent activity against a panel of Gram‐positive bacteria including vancomycin and methicillin resistant *Staphylococcus aureus*. Additionally, the lead compounds show no toxicity against mammalian cells or haemolytic properties at the highest tested concentrations, indicating that the observed activity is specific against bacteria. Finally, these compounds showed no toxicity against *Galleria mellonella* at the highest measured concentrations. However, preliminary efficacy studies in the same animal model found no decrease in bacterial load upon treatment with **Pt1** and **Pt2**. Serum exchange studies suggest that these compounds exhibit high serum binding which reduces their bioavailability *in vivo*, mandating alternative administration routes such as e. g. topical application.

## Graphical abstract

**Metalloantibiotics**: Platinum complexes are mostly known for their anticancer properties, most famously in the drug cisplatin. Herein we describe the high antibacterial properties of platinum cyclooctadiene (COD) complexes. Some of these complexes display MIC values in the nanomolar range while manifesting no toxicity *in vitro* and *in vivo*.

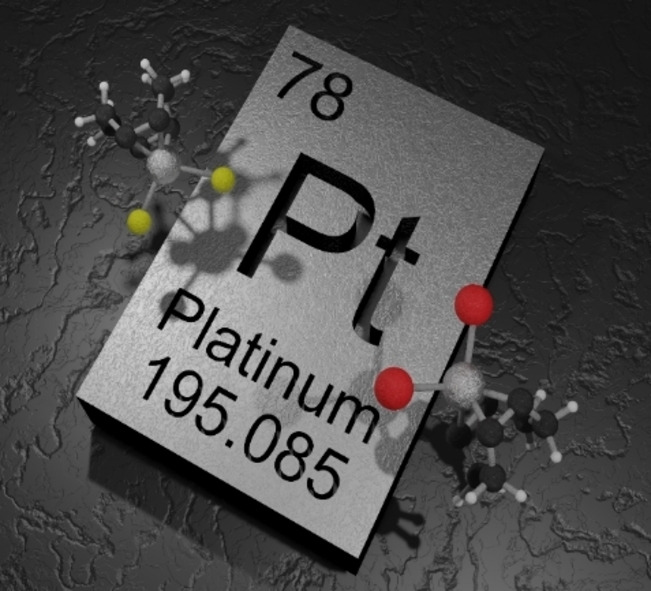

## Introduction

The rapid emergence and spread of multi‐drug resistant bacteria coupled with a severe lack of new and effective antibiotics in the drug development pipeline create an enormous need for innovative approaches.^[1]^ Most medicinal chemistry efforts in the last three decades have followed guidelines that have emerged from years of experience, such as Lipinski's rule of five, leading to strong bias towards lipophilic and flat molecules.^[2]^ However antibiotic molecules rarely conform to these rules and more often than not fly against the intuition of medicinal chemistry paradigms.^[3]^ It is therefore perhaps not all that surprising that the drug pipeline for new antibiotics contains fewer than 50 molecules at the various stages of clinical development, with an even smaller number representing new compound classes and none effective against critical Gram‐negative bacteria.[^1a^, ^1b^, ^1c^]

Metal complexes are relatively rare as drugs, having made their clinical debut in 1978 with the approval of the anticancer drug Cisplatin, which marked the birth of the field of modern inorganic medicinal chemistry. Since then, many groups have studied metal complexes for medicinal applications, but most are still predominantly focused on anticancer applications: in 2020, 12 metal‐based compounds were in clinical trials for this indication.^[4]^

While metal complexes, particularly cisplatin and thousands of its derivatives, have been scrutinized thoroughly as anticancer therapies,^[5]^ few reports have investigated the antimicrobial capabilities of metal‐containing compounds.^[6]^ Interestingly, it was already observed by Rosenberg *et al*. in 1966, that platinum(IV) complexes inhibited the growth of *Escherichia coli*.^[7]^ However, probably since infections were largely effectively controlled by existing antibiotics, attention shifted to the then more urgent search for new anticancer treatments. Nearly half a century later, in 2014, the group of Lippard reported that monofunctional platinum(II) complexes slowed the growth of *E. coli*, induced bacterial filamentation and initiated lysis in lysogenic bacteria.^[8]^ Only a handful of additional papers have since described platinum compounds with any antibacterial effects.^[9]^

In general, metal complexes have only recently gathered attention in the antimicrobial field with multiple reports describing several classes of compounds with promising *in vitro* and *in vivo* results.^[10]^

We recently reported the largest systematic study on the antimicrobial potential of metal‐based compounds to date, showing that metal complexes show a 10‐fold higher hit‐rate against critical ESKAPE bacterial pathogens and fungi compared to organic molecules.^[11]^ Of the 906 metal complexes that were examined in this work, 63 contained platinum. Of these, 27 (43 %) showed activity against at least one of the bacterial and fungal strains tested. Further evaluation of cytotoxicity and haemolysis revealed a total of 18 platinum compounds that possessed antimicrobial activity, with no toxic effects against mammalian cells at the same concentration. In fact, platinum was the element with the largest absolute number of non‐toxic actives in the whole dataset. Based on these results we decided to further investigate the most abundant class of compounds in this set, the organometallic 1,5‐cyclooctadiene (COD) platinum complexes. Pt(COD)‐type complexes have been known and used in the field of organometallic chemistry for decades, generally as synthons for other compounds.^[12]^ They have also found applications in catalysis and materials sciences.^[13]^

The groups of Klein,^[14]^ Ott,^[15]^ and ourselves^[16]^ have further reported on promising anticancer properties of this class of compounds, in particular ones bearing alkyl ligands, which put them in line with the traditional platinum anticancer drugs. However, for antimicrobial compounds, cytotoxicity against any mammalian cells, cancerous or not, is generally an undesired property. We were hence apprehensive at first, when these kinds of platinum compounds displayed good antibacterial and antifungal properties in the screenings performed by the Community for Open Antimicrobial Drug Discovery (CO‐ADD).^[17]^ However, upon closer inspection of the potential toxicity of these compounds we discovered that some compounds were indeed highly antimicrobial while being non‐toxic to human cells at the concentrations tested, accompanied by a lack of toxicity when tested in an *in vivo* model. Herein we report on the antibacterial properties of 14 platinum(COD) complexes, their broad Gram‐positive activity spectrum and preliminary *in vivo* toxicity and efficacy data in the wax moth larvae model *Galleria mellonella*.

## Results and Discussion

**Figure 1:**
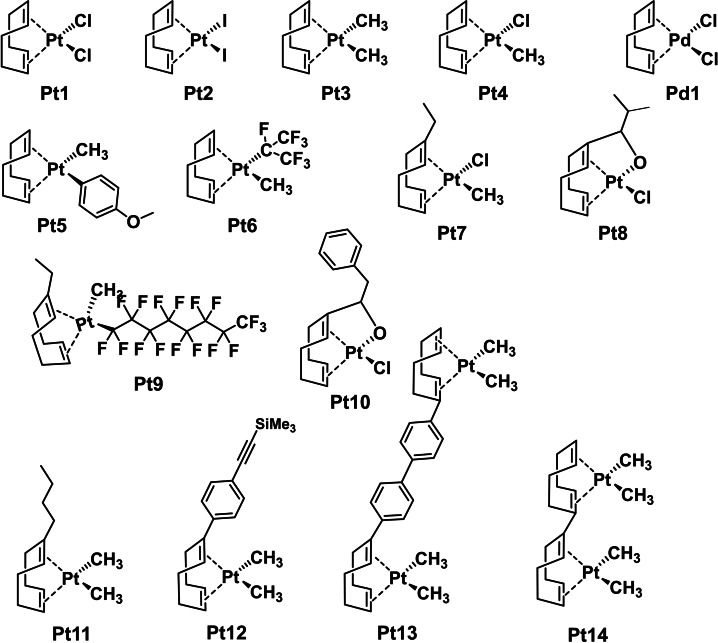
Structures of the platinum and palladium compounds described in this work.

Pt(COD) complexes are widely used as precursors for other platinum compounds, hence their synthesis has been widely documented.^[18]^ The compounds described in Figure 1 were all prepared according to previously published methods, complexes **Pt3‐Pt11** were also reported previously by us and others.[^16^, ^19^] **Pt1**‐**Pt4** and **Pd1** are commercially available from different vendors.

As can be seen in Figure 1, the platinum COD structures in the CO‐ADD database display a variety of features, from simple symmetric and asymmetric compounds to more complex ones that involve multi‐step syntheses. Different substituents on the COD ligand are present (**Pt7‐14**), including coordinating ones (**Pt8**, **Pt10**). Two dinuclear complexes are also present, with varying linkers (**Pt13**, **Pt14**). These compounds were submitted to CO‐ADD as a batch, with no opportunity to pursue iterative structure‐activity relationship studies so the set of compounds in this study represents a random but structurally diverse batch of this particular organometallic compound class.

All compounds submitted to CO‐ADD are initially screened against five bacterial strains (*Staphylococcus aureus* (MRSA), *E. coli*, *Klebsiella pneumoniae, Pseudomonas aeruginosa*, A*cinetobacter baumannii*) and two fungal strains (*Candida albicans*, *Cryptococcus neoformans*) at a single concentration (32 μg/mL or 20 μM) performed as a high‐throughput primary screening.[^11^, ^17a^] Any compounds that show significant growth inhibition against any of the tested strains at this step are then evaluated in a dose‐response assay against these same strains. Table 1 shows the minimum inhibitory concentrations (MIC) of all compounds against this initial panel (of note, **Pt3** and **Pd1** were not initially screened through CO‐ADD but were added to later screens). Remarkably, almost half of the compounds showed some activity against the two yeasts, with **Pt1**‐**2** and **Pt8**‐**9** displaying MIC values in the low micromolar range. **Pt1** and **Pt2** were also the best‐performing compounds against the Gram‐positive methicillin resistant *S. aureus* strain (MRSA) that was tested. The antifungal properties of this compound class are currently being further investigated and will be reported elsewhere. It is notable that the structurally simplest compounds seem to possess the best antibacterial properties. It is also only the symmetric, bis‐halogenated compounds that display these properties.

**Table 1:** Antimicrobial activity (MIC [μM]), toxicity [μM] and haemolytic properties for **Pt1‐14** and **Pd1** determined in the CO‐ADD screening.

	Sa^[a]^	Ec^[b]^	Kp^[c]^	Pa^[d]^	Ab^[e]^	Ca^[f]^	Cn^[g]^	HEK‐293 CC_50_	Haemolysis	
									HC_10_	HC_50_
**Pt1**	0.625–2.5	>20	>20	>20	>20	10–20	1.25–5	>100	>100	>100
**Pt2**	1.25	>20	>20	>20	>20	10	2.5	>100	>100	>100
**Pt3**	n.d.	n.d.	n.d.	n.d.	n.d.	n.d.	n.d.	>100	>100	>100
**Pt4**	>20	>20	>20	>20	>20	5–10	10–20	>100	10.23	52.11
**Pt5**	>20	>20	>20	>20	>20	10	20	>100	88.43	>100
**Pt6**	10	>20	>20	>20	>20	20	20	>100	>100	>100
**Pt7**	>20	>20	>20	>20	>20	10	20	>100	10.27	52.12
**Pt8**	>20	>20	>20	>20	>20	10	1.25	>100	>100	>100
**Pt9**	>20	>20	>20	>20	>20	>20	1.25	>100	>100	>100
**Pd1**	n.d.	n.d.	n.d.	n.d.	n.d.	n.d.	n.d.	>100	7.76	10.14
**Pt10‐14**	>20	>20	>20	>20	>20	–	–	–	–	–
**Colistin^h^**		0.125	0.25	0.25	0.25					
**Vancomycin^h^**	1									
**Tamoxifen**								34.75–30.55		
**Melittin**									1.15	1.82

[a] Sa – *Staphylococcus aureus* ATCC 43300 (MRSA); [b] Ec – *Escherichia coli* ATCC 25922; [c] Kp – *Klebsiella pneumoniae* ATCC 700603 (ESBL SHV‐18); [d] Pa – *Pseudomonas aeruginosa* ATCC 27853; [e] Ab – *Acinetobacter baumannii* ATCC 19606; [f] Ca – *Candida albicans* ATCC 90028; [g] Cn – *Cryptococcus neoformans H99* ATCC 208821; [h] MIC for Colistin and Vancomycin given in μg/mL. MIC – Minimum Inhibitory Concentration; CC_50_ – concentration causing 50 % cytotoxicity; HC_10_, HC_50_ – concentration inducing 10 % or 50 % haemolysis, respectively.

As part of the CO‐ADD screening, all compounds that show any activity against a microbial strain are evaluated for their cytotoxicity against human embryonic kidney cells (HEK‐293) and for haemolysis against human red blood cells. None of the compounds tested for cytotoxicity (**Pt1**‐**9** and **Pd1**) caused any significant cell death up to concentrations of 100 μM. Notably, **Pt4** was reported to display significant cytotoxicity against HT‐29 colon carcinoma (IC_50_=8.3±3.0 μM) and MCF‐7 breast cancer cells (IC_50_=11.2±1.4 μM) by Butsch *et al*.^[14a]^ We have also observed higher IC50 values for the methylated complexes **Pt3** and **Pt4** against HeLa cells in previous work.^[16]^ These results indicate that some of these compounds seemingly display higher toxicity against cancerous cell lines compared to the non‐cancerous HEK‐293 cells. Four compounds caused haemolysis (**Pt4**, **Pt5**, **Pt7**, and **Pd1**). It is noteworthy that **Pt4**, **Pt7** and **Pd1** are all asymmetrically substituted complexes bearing a chloride and a methyl ligand, possibly indicating that this substitution pattern is conducive to haemolytic properties. Neither **Pt1** nor **Pt2** showed any signs of detrimental properties, displaying no detectable cytotoxicity and haemolysis at the highest tested concentrations.

With compounds **Pt1**‐**Pt9** and **Pd1** showing promising properties against Gram‐positive bacteria and/or fungi we tested these compounds against a panel of 12 Gram‐positive bacteria including *S. aureus, Staphylococcus epidermidis, Enterococcus faecium, Enterococcus faecalis, and Bacillus subtilis*. With some exceptions the previous trends were confirmed (Table 2 and Table S1). **Pt1** and **Pt2** displayed the broadest and most pronounced activity spectrum. Against the panel of *S. aureus* strains showed MIC values as low as 390 nM. Their activity was slightly reduced against the vancomycin resistant strain VRS‐1 but **Pt2** was still seven times more active than the control antibiotic vancomycin. Similarly, enhanced activity was observed against *S. epidermidis* for both **Pt1** and **Pt2**. Somewhat higher MICs were detected against the panel of *Enterococcus* strains. However, in contrast to vancomycin, no loss of activity was observed against the multidrug resistant (MDR) strain or the clinical isolate with VanA resistance, whereas vancomycin did not show any antibacterial activity at the tested concentrations. Lastly, both compounds showed low micromolar MIC values against *B. subtilis*. Overall, the difference in activity between **Pt1** and **Pt2** is practically non‐existent, suggesting that the *presence* of two halogens is important to the activity profile of the compounds, whereas the *identity* of the halogen is not. The presence of other ligands represented in the studied compounds seemed to negatively affect their antibacterial activity. This is somewhat of a concern as it constrains further structure‐activity relationship characterization. However, given the data at hand, exploration of substituents on the COD ligand while maintaining the halogen ligands may provide an avenue to generate new, more active, compounds.

**Table 2:** Antibacterial activity of selected compounds against a panel of Gram‐positive bacteria.

Sp	Strain	Pt1	Pt2	Van^[c]^
Sa	ATCC 25923, MSSA	3.125^[a]^	1.56–3.125^[a]^	0.69
	ATCC 43300, MRSA	0.39^[b]^	0.39–0.78^[b]^	0.35–0.69
	NRS 17, GISA	0.78–0.56^[b]^	0.78–1.56^[b]^	5.52
	VRS 1, VRSA (VanA)	12.5^[a]^	6.25^[a]^	>44.2
				
Se	ATCC 14990, type strain	0.781^[a]^	1.56^[a]^	0.69–1.38
	NRS 60, VISE	3.125^[a]^	6.25^[a]^	2.76
				
Em	ATCC 35667, type strain	25^[a]^	25^[a]^	0.35
	ATCC 51559, MDR, VRE (VanA)	12.5^[b]^	12.5–25^[b]^	>44.2
	Clinical Isolate, VRE (VanA)	25–50^[a]^	25^[a]^	>44.2
				
Es	ATCC 29212, control strain	50–100^[a]^	25–50^[a]^	1.38–2.76
	Clinical Isolate, VRE (VanB)	25^[a]^	12.5–25^[a]^	>44.2
				
Bs	ATCC 6051, type strain	6.25^[b]^	3.125–6.25^[b]^	0.35

Antibacterial activity is displayed as MIC μg/mL. **Sp** – Species; *Sa* – Staphylococcus aureus; *Se* – Staphylococcus epidermidis; *Em* – Enterococcus faecium; *Es* – Enterococcus faecalis; *Bs* – *Bacillus subtilis*; MSSA – methicillin susceptible *S. aureus*; MRSA – methicillin resistant *S. aureus*; MDR – multidrug resistant; GISA – glycopeptide intermediate *S. aureus*; VRSA – vancomycin resistant *S. aureus*; VISE – vancomycin intermediate *S. epidermidis*; VRE – vancomycin resistant *Enterococcus*; VanA and VanB are vancomycin resistance genes. [a] MIC determined with n=4 [b] MIC determined with n=2. [c] Vancomycin stock solution was prepared at 2.56 μg/mL and highest tested concentration was 64 μg/mL (44.2 μM).

Amongst the other compounds, **Pt3**, **Pt4**, **Pt6** and **Pt8** showed limited but selective activity against some *S. aureus* strains (Table S1). Of note, while only low levels of activity were observed against the methicillin‐sensitive *S. aureus* (MSSA) strain, the compounds showed significantly more activity against the resistant strains MRSA and NRS 17 GISA (glycopeptide intermediate *S. aureus*). Generally, no activity was found against the various *Enterococcus* strains with a few exceptions. Further studies into the mode of action of this compound class may help in understanding these peculiar trends in activity.

In Gram‐negative bacteria, the double layer membrane, combined with the presence of an efflux pump array make it extraordinarily challenging for any antibiotic compound to enter the cell. This can lead to cases where a compound will test inactive in a Gram‐negative panel, even though it might show good activity if an impermeable membrane or active export did not prevent it from reaching high enough intracellular concentrations. To investigate this dilemma, mutant strains that lack the suite of efflux pumps or possess a more porous membrane have been developed to investigate the activity of promising compounds under more lenient conditions. We hence tested compounds **Pt1‐Pt9** and **Pd1** against the efflux pump deficient *E. coli tolC* strain as well as the non‐efficient lipid A production mutant *lpxC E. coli* strain (Table S2). None of the compounds tested showed any growth inhibition below 50 μM against these mutant strains. This indicates that the observed activity is selective towards Gram‐positive strains and suggests a mechanism of action involving a particular Gram‐positive cellular target that is not found in Gram‐negative bacteria or mammalian cells. Based on these results, an unselective mechanism causing general cellular toxicity can most likely be ruled out as the mode of action for **Pt1** and **Pt2** compounds.

Selectivity against specific strains can be an asset to an antibiotic. Ideally, a future antibiotic drug arsenal will be made up of a series of drugs that are effective against specific strains. This would avoid the blanket use of broad‐spectrum drugs where possible to slow the spread of resistance that is exacerbated by indiscriminate antimicrobial usage today and is becoming a feasible option with the advent of rapid diagnostics that quickly identify the infecting species.

Due to the excellent antibacterial activity exhibited by **Pt1** and **Pt2** against the Gram‐positive panel and the absence of any toxicity indications *in vitro* with good therapeutic indices (>100 against MRSA), we decided to investigate the *in vivo* properties of these metal complexes.

Following a protocol previously reported by us we used larvae of the greater wax moth *Galleria mellonella* as a model organism for *in vivo* toxicity and efficacy.^[20]^ This low‐cost animal model provides good quality data on both toxicity and efficacy of antimicrobial compounds that has been shown to correlate well with results obtained in more expensive and ethically more demanding mouse models.^[21]^ Briefly, stock solutions of **Pt1** and **Pt2** were prepared in DMSO and diluted to final concentrations of 0.4 mM, 100 μM, 10 μM and 1 μM. Due to solubility limitations higher concentrations could not be evaluated. For each concentration five larvae were injected with 10 μL and monitored for 96 hours for survival and health using the *G. mellonella* Health Index Scoring System.^[22]^ For our tested compounds, all larvae were still alive and fully active after four days, indicating that **Pt1** and **Pt2** did not exhibit any toxicity up to 0.4 mM, which equates to a dosage of 6–7.5 mg/kg (**Pt1**) and 9–11 mg/kg (**Pt2**). For comparison purposes we included cisplatin in these experiments as well (up to 3 mM stock in saline). At 36–45 mg/kg all larvae were killed within 10 days, whereas the larvae survived cisplatin injections equating to 18–23 mg/kg. Given that we were unable to dose **Pt1** and **Pt2** at concentrations as high as cisplatin, we are unable to conclude whether they are more or less toxic in this model, however cisplatin showed no antimicrobial activity in all our assays.

**Figure 2:**
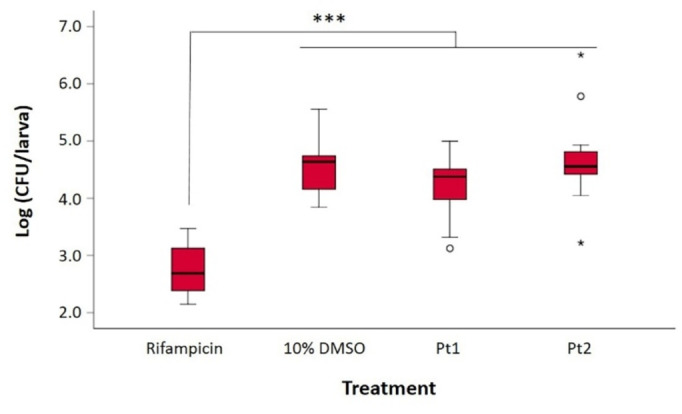
Bacterial load per larva at 24 hours after drug administration. Significant difference was determined by one‐way ANOVA test on log transformed data and Tukey post hoc tests were used for pairwise comparisons (***P<0.001).

With the compounds shown to be tolerated well by *G. mellonella* at these concentrations, we proceeded to conduct preliminary *in vivo* efficacy experiments. Briefly, the larvae were infected with an inoculum of MRSA and two hours later, **Pt1** and **Pt2** were injected at the highest concentration (0.4 mM). Larvae injected with 10 % DMSO and rifampicin were included as controls. Results are represented in Figure 2. Rifampicin had a significant effect on bacterial clearance test in comparison with control (10 % DMSO) and compounds **Pt1** and **Pt2**, causing a 2‐log reduction in CFU. Unfortunately compounds **Pt1** and **Pt2** did not show any significant effect on bacterial clearance at 24 h after drug administration, compared to the control drug.

To obtain some initial insight into the apparent disparity between *in vitro* and *in vivo* results, we repeated the MIC experiments against four selected Gram‐positive strains in the presence of 10 % or 50 % human serum (Table S3). **Pt1** and **Pt2** maintained some antibacterial activity against *B. subtilis* in the presence of 10 % serum, but a complete loss of activity was observed against MRSA and GISA (50 % serum), and VRE (10 % serum). These results suggest that the excellent activity of **Pt1** and **Pt2** observed *in vitro* is almost completely nullified in the presence of serum. Systemic administration is therefore unlikely to be a suitable administration route for this class of compounds unless an alternative formulation that can prevent serum binding (e. g. by lipid or nanoparticle encapsulation) can be found. Nevertheless, topical applications, which do not expose the active compound to blood, could be suitable for Gram‐positive active compounds. For example we have recently reported that cannabidiol (CBD), which also exhibits high serum binding and no systemic efficacy, is able to reduce bacterial load in mouse skin infection models.^[23]^ Further *in vivo* studies should therefore focus on more suitable disease models.

## Conclusion

We have described a new class of antibacterial platinum complexes based on the COD‐ligand scaffold. Of the 14 platinum compounds reported, 10 displayed some antimicrobial activity against Gram‐positive bacteria and/or the yeasts *C. albicans* and *C. neoformans*. Further evaluation of the antifungal properties of this compound class will be reported elsewhere. Interestingly, the complexes with the best antibacterial activity were the simple and symmetric halogen compounds Pt(COD)Cl_2_ (**Pt1**) and Pt(COD)I_2_ (**Pt2**). These two complexes showed excellent antibacterial activity against a broad panel of Gram‐positive strains, with the most pronounced efficacy against *S. aureus*, including MRSA and vancomycin resistant strains, as well as *S. epidermidis* and *B. subtilis*. Despite their resemblance to cisplatin, these compounds did not display any cytotoxicity against human cells or haemolysis in human red blood cells at the highest concentrations tested. The lead compounds **Pt1** and **Pt2** were subsequently tested for toxicity in the moth larvae model *G. mellonella* where no detrimental effects could be detected at the highest injected concentration. Lastly, larvae infected with MRSA were subjected to treatment with **Pt1** and **Pt2**. No significant change in bacterial loads could be observed compared to the DMSO control. Studies of serum reversal effects on MIC values revealed an almost complete loss of the antibacterial activity of **Pt1** and **Pt2** in the presence of human serum. This suggests that serum binding may prevent free platinum complexes from exerting their antibacterial activity in the *in vivo* model. Nevertheless, these compounds could still find potential utility in topical applications such as for skin infections, avoiding exposure to serum.

Our initial set of 14 compounds seems to indicate that modifications to the platinum‐bound ligands have a detrimental effect on their antibacterial activity, reducing the likelihood of successful structure‐activity relationship explorations. However, it could be envisioned, that if the two halogens are maintained as ligands to the platinum, modifications to the COD‐ligand could result in compounds with better properties. At this stage, the mode of action of these compounds is entirely unknown. However, based on the distinct activity profile and the lack of toxicity observed in mammalian cells, a general unspecific toxicity mechanism can most likely be ruled out. The selectivity towards Gram‐positive bacteria points to a specific bacterial cellular target instead, making the compound class interesting for further studies.

There is still a remarkable absence of studies into the antimicrobial properties of metal complexes. While our group and others have begun uncovering the potential of these compounds in recent years, we are still at the beginning of this journey and we encourage other researchers to conduct more thorough and systematic studies. Metal compounds may well become a decisive addition of our future antimicrobial arsenal.

## Experimental Section

The experimental details, including the synthesis and characterisation of **Pt1**‐**Pt14** and **Pd1**, the protocols for the *in vitro* and *in vivo* assays have been described in the Supporting Information.

## Conflict of interest

The authors declare no conflict of interest.

## Supporting information

As a service to our authors and readers, this journal provides supporting information supplied by the authors. Such materials are peer reviewed and may be re‐organized for online delivery, but are not copy‐edited or typeset. Technical support issues arising from supporting information (other than missing files) should be addressed to the authors.

Supporting Information
