# Supplementary material for: Platinum Cyclooctadiene Complexes with Activity against Gram‐positive Bacteria
Source: ChemMedChem. 2021 Jul 8;16(20):3165–71. doi: 10.1002/cmdc.202100157 (PMC8596843; doi:10.1002/cmdc.202100157)
Supplement: Supplementary file 1 — Supporting Information [file CMDC-16-3165-s001.pdf]

ChemMedChem

Supporting Information

Platinum Cyclooctadiene Complexes with Activity against Gram-positive Bacteria

Angelo Frei,* Soumya Ramu, Gabrielle J. Lowe, Hue Dinh, Lucie Semenec, Alysha G. Elliott, Johannes Zuegg, Anke Deckers, Nicole Jung, Stefan Bräse, Amy K. Cain, and Mark A. T. Blaskovich*

Experimental Part

Data availability

The reaction descriptions and analytical datasets of the compounds used in this study described below are publicly available as digital research materials in the repository Chemotion. The data can be accessed on the repositories web access: www.chemotion-repository.net/home or via the DOI-link cited after the respective reaction.

Synthesis, Analysis and References for Compounds Pt1-Pt9, Pd1

Most of the compounds described here were gained in former chemistry research projects and were published before. The performed reactions and obtained characterizations are described as follows. The compounds were used from a library that was hosted for several years at the Molecule Archive at KIT therefore, selected analysis was repeated to approve the identity of the samples that were used in this study. The data obtained can be accessed through the given DOIs, linking to the gained datasets.

General remarks on methods

NMR spectra were recorded on a Bruker Ascend 400 spectrometer as solutions at room temperature. Chemical shifts (δ) are expressed in parts per million (ppm) downfield from tetramethylsilane (TMS). References for 1H NMR and 13C NMR were the residual solvent peaks of chloroform (1H: δ = 7.26 ppm) and d1-chloroform (13C: δ = 77.0 ppm). All coupling constants (J) are absolute values and are expressed in Hertz (Hz). The description of signals includes: s = singlet, d = doublet, t = triplet, m = multiplet, dd = doublet of doublets and ddd = double doublet of doublets and so forth, s d, singlet + doublet (of singlets) due to coupling with 195Pt. The spectra were analyzed according to first order. The assignments of the signal structure in 1H NMR were made by the multiplicity and for 13C NMR by DEPT 90- and DEPT 135-spectra (DEPT = Distortionless Enhancement by Polarization Transfer) and are described as follows: + = primary or tertiary C atom (positive DEPT-signal), – = secondary C-atom (negative signal) and Cq = quaternary C-atom (no signal). IR spectra were recorded on a Bruker IR-ATR spectrometer. The compounds were measured as pure substances by ATR technique (ATR = attenuated total reflection). Mass spectra were measured by EI-MS (electron impact mass spectrometry) and were recorded on a Finnigan MAT 95. The peaks are given as mass-to-charge-ratio (m/z). The molecule peak is given as [M]+ and characteristic fragment peaks are given as [M–fragment]+ or [fragment]+. The signal intensities are given in percent, relatively to the intensity of the base signal (100 %). For the high resolution mass, the following abbreviations were used: Calcd = calculated data, Found = measured data. The software of EI mass spectrometer adds the mass of one electron. Analytical thin layer chromatography (TLC) was carried out on Merck silica gel coated aluminum plates (silica gel 60, F254), detected under UV-light at 254 nm or stained with "Seebach staining solution" (mixture of molybdato phosphoric acid, cerium(IV)-sulfate tetrahydrate, sulfuric acid and water) or basic potassium permanganate solution. Solvent mixtures are understood as volume/volume. Solvents, reagents and chemicals were purchased from Sigma-Aldrich, ABCR, Thermo Fisher, TCI, ChemPur and Acros Organics. All solvents, reagents and chemicals were used as purchased unless stated otherwise. The reactions were carried out under argon atmosphere in oven-dried and previously evacuated glass ware. Liquids were transferred with plastic syringes and steel cannula.

Cycloocta-1,5-diene;dichloroplatinum, PtCl2(COD) (Pt1)

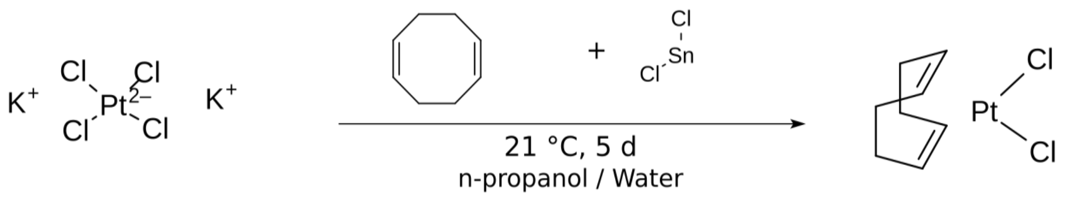

Formula: $C_8H_{12}Cl_2Pt$; Smiles: C1CC=CCCC=C1.Cl[Pt]Cl;
InChIKey: VVAOPCKKNIUEEU-PHFPKPIQSA-L, CAS: 12080-32-9

Dipotassium;tetrachloroplatinum(2-) (111 mg, 267 µmol, 1.00 equiv) was dissolved in water (1.78 mL) and propan-1-ol (1.78 mL) and (1Z,5Z)-cycloocta-1,5-diene (200 mg, 1.85 mmol, 6.90 equiv) was added. Dichlorotin (1.50 mg, 7.91 µmol, 0.0296 equiv) was added and the reaction mixture was stirred at 21 °C for five days until the solution was completely decolorized. The resulting beige solid was filtered off, washed twice with water and once with ethanol and dried in high vacuum. (1Z,5Z)-Cycloocta-1,5-diene;dichloroplatinum (88.4 mg, 236 µmol) was obtained in 88% yield.

^{1}H NMR (400 MHz, $CDCl_3$, ppm) δ = 5.71–5.53 (m, 4H, C*H*), 2.78–2.64 (m, 4H, CH_2), 2.36–2.16 (m, 4H, CH_2); ^{13}C NMR (100 MHz, $CDCl_3$, ppm) δ = 100.1 (+, s, d, ^1JPtC = 153 Hz, 4 × *C*H), 30.9 (+, 4 × *C*H_2); IR (ATR): $\tilde{\nu}$ = 3007 (vw), 1474 (vw), 1449 (vw), 1423 (vw), 1337 (w), 1310 (vw), 1177 (vw), 1087 (vw), 1028 (vw), 1007 (vw), 909 (vw), 870 (w), 830 (w), 809 (vw), 779 (vw), 694 (vw), 581 (vw), 473 (w), 456 (vw) cm^{-1}; MS (70 eV, EI), *m/z* (%): 378/377/376/375/374/373/372 (14/11/65/55/100/85/76) [M^+], 341/340/339/ 338/337 (15/19/44/56/25) [$(M\text{-}Cl)^+$], 302/301/300/299/298 (77/95/90/65/63) [$(M–2×Cl)^+$], 273 (21), 272 (30), 246 (13); ^{195}Pt NMR (129 MHz, $CDCl_3$, ppm): δ = –3330 (s).

Link to selected data that was repeated to approve the identity of the compounds:

https://dx.doi.org/10.14272/reaction/SA-FUHFF-UHFFFADPSC-VVAOPCKKNI-UHFFFADPSC-NUHFF-LOTXU-NUHFF-ZZZ
https://dx.doi.org/10.14272/VVAOPCKKNIUEEU-PHFPKPIQSA-L.1

Cycloocta-1,5-diene;diiodoplatinum, PtI2(COD) (**Pt2**)

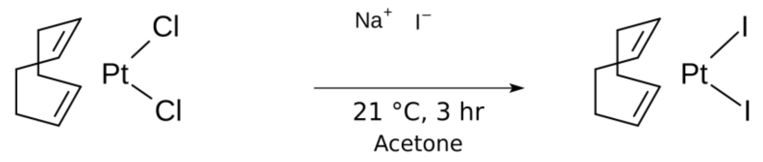

Formula: $C_8H_{12}I_2Pt$; Smiles: C1CC=CCCC=C1.I[Pt]I
InChIKey: SGSFNZOKVYTGRR-PHFPKPIQSA-L, CAS: 12266-72-7

To a solution of (1Z,5Z)-cycloocta-1,5-diene;dichloroplatinum (4.20 g, 11.2 mmol, 1.00 equiv) in acetone (50.0 mL), sodium;iodide (3.62 g, 24.1 mmol, 2.15 equiv) was added at 21 °C. The solution immediately turned yellow and was stirred for three hours. The acetone was removed in vacuo and the obtained residue was washed with water (3 x 50 ml). The precipitate was then dissolved again in dichloromethane (200 mL), the solution was filtered, dried over Na_2SO_4 and the solvent was removed under reduced pressure. After drying, PtI2(COD) (6.20 g, 11.1 mmol) was obtained as a yellow solid in 99% yield.

^{1}H NMR (400 MHz, $CDCl_3$) δ = 5.85–5.68 (m, 4H, CH), 2.55–2.35 (m, 4H, CH2), 1.80–2.04 (m, 4H, CH_2); ^{13}C NMR (100 MHz, $CDCl_3$) δ = 102.48 (+, s d, *J* = 122 Hz, 4xCH), 31.6 (-, 4xCH_2); EI-MS (70 eV) *m/z (%)* = 556/557/558/559/560 (89/100/75/5/18) [M^+], 428/429/430/431 (21/58/79/7), 423/424/425/426/427 (3/5/13/11/27), 300/301/302/303 (15/20/10/8) [M^+-2´HI], 295/296/297/299 (6/12/25/43). HRMS ($C_{10}H_{12}I_2Pt$): Calcd 556.8676; Found 556.8679; IR (ATR, ṽ) = 2997 (w), 2927 (w), 2874 (w), 2822 (w), 1496 (w), 1472 (w), 1446 (w), 1419 (s), 1373 (w), 1337 (m), 1306 (m), 1225 (m), 1174 (m), 1162 (m), 1086 (w), 1068 (m), 996 (vs), 899 (w), 866 (vs), 843 (m), 820 (vs), 798 (s), 775 (vs) cm^{-1}; CHN($C_{10}H_{12}I_2Pt$): Calcd C 17.25, H 2.17; Found C 17.24, H 2.23.

Link to selected data that was repeated to approve the identity of the compounds:

https://dx.doi.org/10.14272/reaction/SA-FUHFF-UHFFFADPSC-SGSFNZOKVY-UHFFFADPSC-NUHFF-LOTXU-NUHFF-ZZZ
https://dx.doi.org/10.14272/SGSFNZOKVYTGRR-PHFPKPIQSA-L.1

(1,5-Cyclooctadiene)dimethylplatinum (II), PtMe2(COD) (**Pt3**)[1]

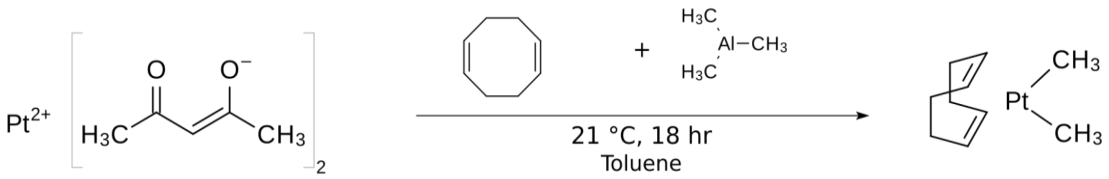

Formula: $C_{10}H_{18}Pt$; Smiles: C1CC=CCCC=C1.C[Pt]C
InChIKey: ZABVBYGUHBRHFJ-PHFPKPIQSA-N, CAS: 12266-92-1

(1Z,5Z)-Cycloocta-1,5-diene (463 mg, 4.28 mmol, 1.10 equiv) and (Z)-4-oxopent-2-en-2-olate;platinum(2+) (1.53 g, 3.89 mmol, 1.00 equiv) were dissolved in absolute toluene (150 mL) under argon in a Schlenk flask and then trimethylalumane (841 mg, 5.84 mL, 11.7 mmol, 2.00 M, 3.00 equiv) was added. The reaction mixture was stirred overnight until the diene could no longer be detected by TLC. The dark yellow to black solution was then quenched with saturated ammonium chloride solution (10 mL). The organic phase was washed with 1 M hydrochloric acid (3 × 25 mL), saturated aqueous sodium chloride solution (25 mL), dried over sodium sulfate and the solvent was removed under reduced pressure. The crude product was purified by column chromatography (cyclohexane/ 2% triethylamine) and $PtMe_2$(COD) (805 mg, 2.42 mmol) was obtained as a colorless solid in 62% yield. R_f = 0.42 (cyclohexane/triethylamine (2%)).

^{1}H NMR (300 MHz, $CDCl_3$, ppm) δ = 4.93–4.69 (m, 4H, C*H*), 2.46–2.19 (m, 8H, C*H*$_2$), 0.95–0.66 (m, 6H, PtC*H*$_3$); 13C NMR (75 MHz, $CDCl_3$, ppm) δ = 99.2 (+, s d, *J* = 53.2 Hz, 4 × *C*H), 30.0 (−, 4 × *C*H2), 4.6 (+, s d, *J* = 772 Hz, 2 × Pt*C*H3); MS (70 eV, EI), *m/z* (%): 336/334/333/332 (5/21/27/24) [M+], 320/319/318/317/316 (8/68/87/100/27) [M+–CH3], 304/303/302/301/300 (30/46/47/31/31) [M+–2 × CH3], 284 (4), 274 (11), 272 (18), 271 (14), 248 (11), 247 (11), 105 (4), 91 (5), 79 (5), 43 (4). HRMS (C10H18): Calcd 333.1056; Found 333.1055; IR (ATR) $\tilde{\nu}$ = 2961 (vw), 2919 (vw), 2866 (w), 2791 (vw), 1518 (w), 1474 (vw), 1425 (w), 1339 (vw), 1259 (m), 1227 (vw), 1177 (vw), 1086 (vw), 1014 (m), 978 (vw), 858 (w), 795 (m), 687 (w), 562 (w), 544 (m), 451 (w).

Link to selected data that was repeated to approve the identity of the compounds:

https://dx.doi.org/10.14272/reaction/SA-FUHFF-UHFFFADPSC-ZABVBYGUHB-UHFFFADPSC-NUHFF-NOTXU-NUHFF-ZZZ
https://dx.doi.org/10.14272/ZABVBYGUHBRHFJ-PHFPKPIQSA-N.1

Chloro(1,5-cyclooctadiene)methylplatinum, PtMeCl(COD) (**Pt4**)[1]

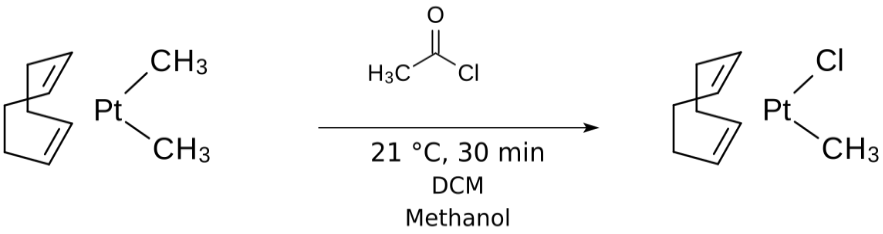

Formula: $C_9H_{15}ClPt$; Smiles: C1CC=CCCC=C1.C[Pt]Cl

InChIKey: UFZABDNBGQRIHA-PHFPKPIQSA-M, CAS: 50978-00-2

To a solution of PtMe2(COD) (536 mg, 1.61 mmol, 1.00 equiv) in methylene chloride (5.70 mL) and methanol (3.80 mL), acetyl chloride (126 mg, 1.61 mmol, 1.00 equiv) was added at 21 °C. After complete addition of the acetyl chloride, the reaction mixture was stirred for 10 minutes and the volume was then reduced to 2 mL. The resulting colorless crystals were cooled overnight, then filtered and washed with a small volume of pentane. After drying, PtMeCl(COD) (556 mg, 1.57 mmol) was obtained as a colorless solid in 98% yield.

^{1}H NMR (300 MHz, $CDCl_3$, ppm) δ = 5.51 (m, 2H, C*H*), 4.49 (m, 2H, C*H*), 2.60–2.14 (m, 8H, CH_2), 0.90 (s d, $^2J_{PtH}$ = 71 Hz, 3H, CH_3); 13C NMR (75 MHz, $CDCl_3$, ppm) δ = 113.5 (+, s d, 1*J*PtC = 30.8 Hz, 2 × *C*H), 83.6 (+, s d, 1*J*PtC = 216 Hz, 2 × *C*H), 31.8 (–, s d, 2*J*PtC = 12.2 Hz, 2 × *C*H2), 28.1 (–, s d, 2*J*PtC = 11.7 Hz, 2 × *C*H2), 5.3 (+, s d+, 1*J*PtC = 613 Hz, Pt*C*H3); MS (70 eV, EI), *m/z* (%): 356/355/354/353/352 (16/15/43/39/33) [M+], 341/340/339/338/337 (29/25/74/71/66) [(M–CH3)+], 303/302/301/300/299 (83/63/100/45/67) [(M—Cl–CH3)+]. HRMS (C9H15ClPt): Calcd 353.0510; Found 353.0510; IR (ATR): $\tilde{\nu}$ = 3001 (vw), 2945 (vw), 2879 (w), 2793 (vw), 1546 (vw), 1475 (w), 1423 (w), 1178 (vw), 992 (w), 861 (w), 691 (w), 552 (w) cm^{-1}; 195Pt NMR (129 MHz, $CDCl_3$, ppm) δ = –3496 (s).

Link to selected data that was repeated to approve the identity of the compounds:

https://dx.doi.org/10.14272/reaction/SA-FUHFF-UHFFFADPSC-UFZABDNBGQ-UHFFFADPSC-NUHFF-MOTXU-NUHFF-ZZZ
https://dx.doi.org/10.14272/UFZABDNBGQRIHA-PHFPKPIQSA-M.1

Bis(chloranyl)palladium;(1Z,5Z)-cycloocta-1,5-diene, $PdCl_2$(COD) (**Pd1**)

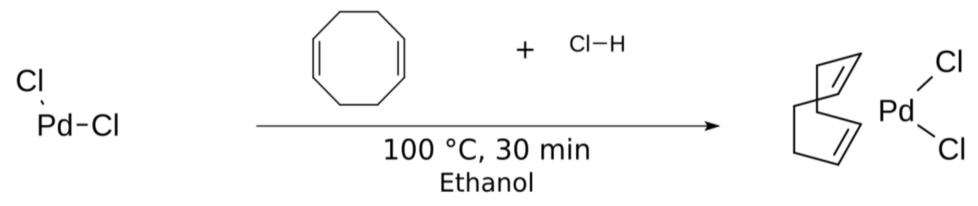

Formula: $C_8H_{12}Cl_2Pd$; Smiles: C1CC=CCCC=C1.Cl[Pd]Cl
InChIKey: RRHPTXZOMDSKRS-PHFPKPIQSA-L, CAS: 12107-56-1

Palladium(II) chloride (200 mg, 1.13 mmol, 1.00 equiv) was dissolved in 0.5 mL of concentrated HCl under reflux. The cold solution was diluted in ethanol (17.0 mL) and filtered. While stirring, (1Z,5Z)-cycloocta-1,5-diene (264 mg, 2.44 mmol, 2.16 equiv) was added to the solution. A yellow solid was formed, after another 10 minutes of stirring, the solid was filtered off and washed with diethyl ether (3 × 3 mL). After drying, bis(chloranyl)palladium;(1Z,5Z)-cycloocta-1,5-diene (308 mg, 1.08 mmol) was obtained as yellow solid in 96% yield.

^{1}H NMR (400 MHz, $CDCl_3$, ppm) δ = 6.31 (bs, 4H, C*H*), 2.88–2.94 (m, 4H, CH_2), 2.53–2.60 (m, 4H, CH_2); ^{13}C NMR (100 MHz, $CDCl_3$, ppm) δ = 116.6 (+, 4 x *C*H), 31.0 (-, 4 x *C*H_2); IR (ATR, $\tilde{\nu}$) = 3053 (w), 2956 (s), 2919 (s), 2868 (s), 2140 (w), 2132 (w), 1601 (vs), 1533 (w), 1466 (vs), 1443 (s), 1421 (s), 1384 (vs), 1377 (vs), 1323 (m), 1276 (w), 1210 (s), 1156 (w), 1112 (w), 1086 (m), 1057 (s), 1028 (s), 1001 (m), 864 (vs), 830 (vs), 803 (vs), 765 (m), 732 (m), 691 (m), 666 (m), 633 (m), 560 (s), 545 (vs), 537 (vs), 524 (vs), 510 (vs), 497 (vs), 477 (vs), 460 (vs), 452 (vs), 442 (vs), 438 (vs), 428 (vs), 402 (vs), 388 (vs), 377 (vs) cm^{-1}.

Link to selected data that was repeated to approve the identity of the compounds:

https://dx.doi.org/10.14272/reaction/SA-FUHFF-UHFFFADPSC-RRHPTXZOMD-UHFFFADPSC-NUHFF-LOTXU-NUHFF-ZZZ
https://dx.doi.org/10.14272/RRHPTXZOMDSKRS-PHFPKPIQSA-L.1

[(1,2,5,6-η)-1,5-Cyclooctadiene]methyl(4-methoxyphenyl)platinum, PtMe(C4H4OMe)(COD) (Pt5)[2]

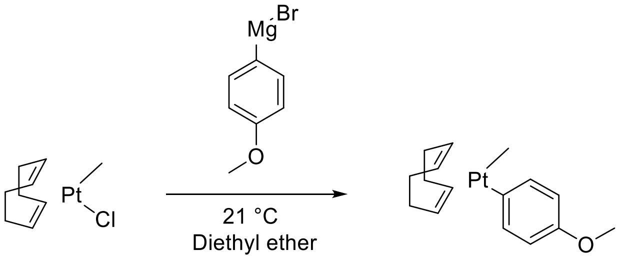

Formula: $C_{16}H_{22}OPt$; Smiles: C1CC=CCCC=C1.COc1ccc(cc1)[Pt]C
InChIKey: MZKVZKDVJWCKKC-PHFPKPIQSA-N, CAS: 1163143-44-9

To a solution of PtMeCl(COD) (200 mg, 565 µmol, 1.00 equiv) in diethyl ether (15.0 mL) was added (4-methoxyphenyl)magnesium bromide (143 mg, 339 µL, 678 µmol, 2.00M, 1.20 equiv) at 21 °C. The reaction mixture was stirred for two hours and then quenched with saturated aqueous ammonium chloride solution. The aqueous phase was extracted with diethyl ether and dried over magnesium sulfate. The solvent was removed under reduced pressure. The obtained crude product was purified via flash-chromatography on silica gel using cyclohexane/ethyl acetate 10:1. After drying, PtMe(C4H4OMe)(COD) (125 mg, 294 µmol) was obtained as a orange solid in 52% yield. R_f = 0.52 (cyclohexane/ethyl acetate 10:1).

1H NMR (400 MHz, CDCl3, ppm) δ = 7.24–7.20 (m, 2H, H_{ar}), 6.79–6.75 (m, 2H, Har), 5.06 (m, 2H, CH), 4.87 (m, 2H, CH), 3.76 (m, 3H, CH_3), 2.44 (m, 8H, CH_2), 0.85 (s d, $^2J_{PtH}$ = 82 Hz, 3H, OCH_3). Impurities: spectrum contains ca. 15% impurities visible in the aromatic region; ^{13}C NMR (100 MHz, CDCl3, ppm) δ = 156.0 ($C_{quart.}$), 148.2 ($C_{quart.}$), 134.8 (+, s d, 42.4 Hz, CH), 113.5 (+, s d, J = 80.1 Hz, CH), 101.8 (+, s d, J = 50.9 Hz, CH), 101.3 (+, s d, J = 52.4 Hz, CH), 54.9 (+, CH_3), 29.7 (–, 4xCH_2), 30.1 (–, 4xCH_2), 6.66 (+, CH_3); MS (70 eV, EI) m/z (%) = 425/424 (41/31) [M+], 304/303/303/302/301300/299298/297 (70/100/98/48/46/60/50/54) [M+–C_6H_4OMe–Me]. HRMS (C16H22OPt): Calcd 425.1313; Found 425.1316; IR (KBr): 3062 (vw), 2998 (w), 2919 (w), 2820 (w), 1605 (vw), 1582 (w), 1481 (m), 1264 (m), 1228 (m), 1171 (m), 1032 (m), 799 (m), 586 (w), 550 (w), 523 (w), 447 (w) cm^{-1}.

Link to selected data that was repeated to approve the identity of the compounds:
https://dx.doi.org/10.14272/reaction/SA-FUHFF-UHFFFADPSC-MZKVZKDVJW-UHFFFADPSC-NUHFF-NOTXU-NUHFF-ZZZ
https://dx.doi.org/10.14272/MZKVZKDVJWCKKC-PHFPKPIQSA-N.1

[(1,2,5,6-η)-1,5-Cyclooctadiene]methyl[1,2,2,2-tetrafluoro-1-(trifluoromethyl)ethyl]platinum, PtMe(iC3F7)(COD) (Pt6)[1,3,4]

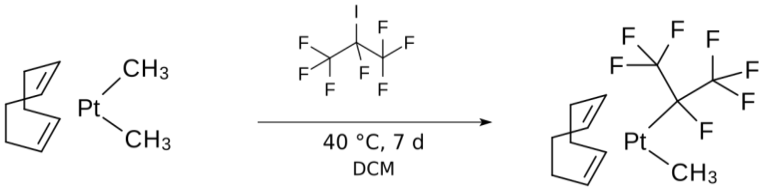

Formula: $C_{12}H_{15}F_7Pt$; Smiles: C1CC=CCCC=C1.C[Pt]C(C(F)(F)F)(C(F)(F)F)F
InChIKey: FVXGDJGGNGUZCN-PHFPKPIQSA-N, CAS: 887305-17-1

$PtMe_2$(COD) (300 mg, 900 µmol, 1.00 equiv) was dissolved in abs. methylene chloride (4.00 mL) at 21 °C and 1,1,1,2,3,3,3-heptafluoro-2-iodopropane (4.00 g, 13.5 mmol, 15.0 equiv) was added. The reaction mixture was stirred at 35–40 °C for seven days in the dark. For work up the solvent was removed under reduced pressure. The crude product was purified by column chromatography on silica gel (cyclohexane/ ethyl acetate 30:1) and PtMe(iC3F7)(COD) (396 mg, 813 µmol) obtained as a colourless solid in 90% yield. R_f = 0.44 (cyclohexane/ethyl acetate 5:1).

^{1}H NMR (400 MHz, $CDCl_3$, ppm) δ = 5.52 (s d, ^2JPtH = 31 Hz, 2H, C*H*), 4.97 (s d, ^2JPtH = 31 Hz, 2H, C*H*), 2.55–2.30 (m, 8H, CH_2), 0.79 (s d, ^2JPtH = 79 Hz, 3H, PtCH_3); 19F NMR (400 MHz, $CDCl_3$, ppm) δ = –183.6–(–183.1) (m, 1 F, C*F*(CF_3)$_2$), –68.4 (td, 6 F, 3*J*PtF = 51 Hz, 2*J*FF = 9.1 Hz, CF_3); MS (70 eV, EI) *m/z (%)* = 486/487/488/489/490 (9/11/8/1/1) [M+], 471/472/473/474/475 (7/8/6/1/1) [(M-CH3)+], 321/322/323/324/325 (79/65/48/3/12) [(M-C4F8-CH3)+], 317/318/319/320 (94/100/83/10) [(M-C4F9)+], 300/301/302/303/304 (25/ 37/22/20/4) [(M-C4F9-CH4)+], 295/296/297/298/299 (7/10/23/15/35). HRMS (C12H15F7Pt): Calcd 487.0710; Found 487.0707; IR (ATR): ṽ = 2902 (w), 2840 (w), 1488 (w), 1430 (m), 1266 (m), 1206 (m), 1172 (m), 1109 (w), 1089 (m), 997 (w), 910 (w), 898 (w), 866 (w), 701 (w) cm^{-1}; ^{195}Pt NMR (129 MHz, $CDCl_3$, ppm): δ = –3764 (s).

Link to selected data that was repeated to approve the identity of the compounds:
https://dx.doi.org/10.14272/reaction/SA-FUHFF-UHFFFADPSC-FVXGDJGGNG-UHFFFADPSC-NUHFF-NOTXU-NUHFF-ZZZ
https://dx.doi.org/10.14272/FVXGDJGGNGUZCN-PHFPKPIQSA-N.1

Chloro[(1,2,5,6-η)-1-ethyl-1,5-cyclooctadiene]methylplatinum, PtMeCl(Et-COD) (**Pt7**)[1]

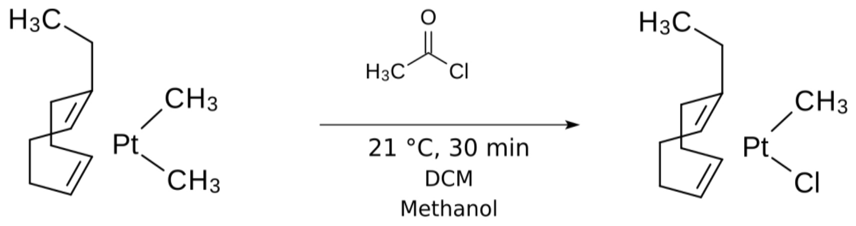

Formula: $C_{11}H_{19}ClPt$; Smiles: CCC1=CCCC=CCC1.C[Pt]Cl
InChIKey: UMUREYUUCIUYJA-RXRNHWPWSA-M, CAS: 1620985-78-5

Acetyl chloride (61.3 mg, 780 µmol, 1.00 equiv) was added to a solution of $PtMe_2$(Et-COD) (282 mg, 780 µmol, 1.00 equiv) in methylene chloride (2.00 mL) and methanol (1.20 mL) at 21 °C. After complete addition of the acetyl chloride, the reaction mixture was stirred for 10 minutes and the volume was then reduced to 1 mL. The resulting colorless crystals were cooled overnight, then filtered and washed with a little amount of pentane. After drying, PtMeCl(Et-COD) (186 mg, 487 µmol) was obtained as colourless crystals in 62% yield.

^{1}H NMR (400 MHz, $CDCl_3$, ppm) δ = 5.42–5.31 (m, 1H, C*H*), 4.51–4.24 (m, 2H, C*H*), 2.71–1.99 (m, 10H, CH_2), 1.12 (t, ^3JHH = 7.4 Hz, 3H, CH_3), 0.87 (s d, ^2JPtH = 71 Hz, 3H, PtCH_3); 13C NMR (100 MHz, $CDCl_3$, ppm) δ = 137.2 (*C*quart, s d, 1*J*PtC = 16 Hz), 109.6 (+, s d, 1*J*PtC = 13 Hz, *C*H), 82.5 (+, s d, 1*J*PtC = 109 Hz, *C*H), 81.9 (+, s d, 1*J*PtC = 114 Hz, *C*H), 33.8 (–, *C*H2), 33.1 (–, *C*H2), 29.9 (–, *C*H2), 29.5 (–, *C*H2), 29.3 (–, *C*H2), 13.7 (+, s d, *J* = 11.6 Hz, *C*H3), 3.2 (+, s d, *J* = 614 Hz, Pt*C*H3); MS (70 eV, EI), *m/z* (%): 380/381/382/383/384 (8/10/10/4/4) [(M–CH3)+], 365/366/367/368/369 (18/32/9/7/9) [(M–C2H5)+], 327/328/329/330/331 (44/50/100/78/56) [(M–Cl–CH3)+]. HRMS (C11H19ClPt), [(M–CH3)+]: Calcd 381.0818; Found 381.0817; IR (ATR): ṽ = 2959 (w), 2925 (w), 2876 (w), 2835 (vw), 1563 (vw), 1480 (vw), 1459 (w), 1426 (w), 1372 (vw), 1340 (w), 1318 (w), 1239 (w), 1219 (w), 1177 (vw), 1108 (vw), 1057 (w), 1038 (w), 1019 (w), 1004 (w), 980 (w), 933 (w), 911 (w), 863 (w), 829 (w), 790

(w), 737 (vw), 622 (w), 565 (w), 530 (w), 416 (w) cm^{-1}; 195Pt NMR (129 MHz, CDCl3, ppm) δ = –3471 (s).

Link to selected data that was repeated to approve the identity of the compounds:
https://dx.doi.org/10.14272/reaction/SA-FUHFF-UHFFFADPSC-UMUREYUUCI-UHFFFADPSC-NUHFF-MLLUO-NUHFF-ZZZ
https://dx.doi.org/10.14272/UMUREYUUCIUYJA-RXRNHWPWSA-M.1

(1-((1E,5Z)-Cycloocta-1,5-dien-1-yl)-2-methylpropoxy)platinum(II)-chloride (Pt8)[v]

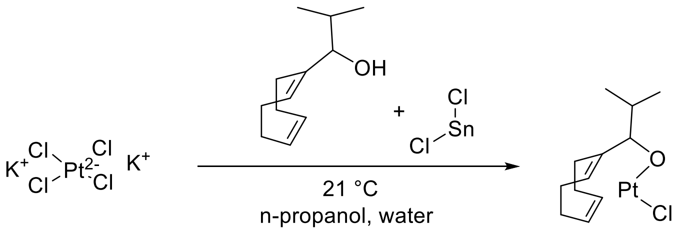

Formula: $C_{12}H_{19}ClOPt$; Smiles: Cl[Pt]OC(C1=CCCC=CCC1)C(C)C
InChIKey: QPCHCPTWJXRVTG-DTPNCXJISA-M, CAS: 2197920-14-0

Dipotassium;tetrachloroplatinum(2-) (199 mg, 479 µmol, 1.00 equiv) was dissolved in water and stirred at 21 °C. 1-[(1E,5Z)-cycloocta-1,5-dien-1-yl]-2-methyl-propan-1-ol (596 mg, 3.31 mmol, 6.90 equiv) was dissolved in *n*-propanol and added to the solution using a glass pipette. Finally, dichlorotin (2.72 mg, 14.4 µmol, 0.030 equiv) was added and the reaction mixture was stirred at 21 °C for one to six days until the solution was completely colorless. The resulting solid was filtered and washed twice with water and once with a little amount of ethanol. The solid was dried under vacuum. The crude product was recrystallized from methanol. (1-((1E,5Z)-Cycloocta-1,5-dien-1-yl)-2-methylpropoxy)platinum(II)-chloride (147 mg, 359 µmol) was obtained as a colorless solid in 75% yield.

^{1}H NMR (400 MHz, $CDCl_3$, ppm) δ = 5.81–5.55 (m, 2H, C*H*COD), 5.38 (dd, ^3J = 7.1, ^4J = 3.1 Hz, 1H, C*H*COD), 3.80 (d, ^3J = 9.7 Hz, 1H, C*H*OPt), 2.93–2.59 (m, 5H, C*H*(CH_3)$_2$, 2 × CH_2), 2.44–2.25 (m, 1H, CH_2), 2.16–2.10 (m, 1H, CH_2), 1.91 (dt, ^3J = 14.6, 7.6 Hz, 1H, CH_2), 1.85–1.74 (m, 1H, CH_2), 1.02 (d, ^3J = 6.4 Hz, 3H, CHCH_3), 0.56 (d, ^3J = 6.8 Hz, 3H, CHCH_3); 13C NMR (100 MHz, $CDCl_3$, ppm) δ = 127.4 (*C*q), 101.1 (+, *C*H), 100.0 (+, *C*H), 95.9 (+, *C*H), 82.2 (*C*HOPt), 31.9 (+, *C*H(CH3)2), 31.3 (−, *C*H2), 30.9 (−, *C*H2), 30.3 (−, *C*H2), 29.5 (−, *C*H2), 20.2 (+, CH3), 19.1 (+, *C*H3). MS (70 eV, EI), *m/z* (%): 409 (20) [M+], 374/373/372 (82/99/100) [C12H19OPt+]. HRMS (C12H19OClPt): Calcd 409.0767; Found 409.0765; IR (ATR): $\tilde{\nu}$ = 3504 (vw), 2956 (w), 1459 (vw), 1421 (vw), 1384 (vw), 1364 (w), 1338 (vw), 1315 (w), 1238 (vw), 1168 (vw), 1117 (vw), 1051 (vw), 1024 (w), 952 (vw), 905 (vw), 885 (w), 866 (w), 866 (w), 827 (w), 804 (vw), 769 (vw), 732 (vw), 691 (vw), 636 (w), 571 (vw), 529 (vw), 479 (w), 463 (w), 416 (m) cm^{-1}; ^{195}Pt NMR (129 MHz, $CDCl_3$, ppm) δ = −3333.

Link to selected data that was repeated to approve the identity of the compounds: https://dx.doi.org/10.14272/reaction/SA-FUHFF-UHFFFADPSC-QPCHCPTWJX-UHFFFADPSC-NUHFF-MAJTN-NUHFF-ZZZ.1
https://dx.doi.org/10.14272/QPCHCPTWJXRVTG-DTPNCXJISA-M.3

[(1,2,5,6-)-1Eethyl-1,5-cyclooctadiene](heptadecafluorooctyl)methylplatinum, PtMe(nC8F17)(Et-COD) (Pt9)[1]

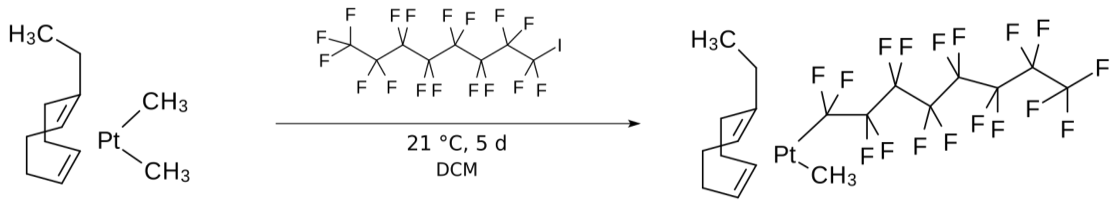

Formula: $C_{19}H_{19}F_{17}Pt$; Smiles:
C[Pt]C(C(C(C(C(C(C(C(F)(F)F)(F)F)(F)F)(F)F)(F)F)(F)F)(F)F)(F)F.CCC1=CCCC=CCC1
InChIKey: ARCQLLPCVYIECO-RXRNHWPWSA-N, CAS: 1620985-75-2

PtMe2(Et-COD) (200 mg, 553 µmol, 1.00 equiv) was dissolved in abs. methylene chloride (1.00 mL) at 21 °C and 1,1,1,2,2,3,3,4,4,5,5,6,6,7,7,8,8-heptadecafluoro-8-iodooctane (1.51 g, 2.77 mmol, 5.00 equiv) was added. The reaction mixture was stirred for five days at 21 °C. The crude product was purified by column chromatography on silica gel (cyclohexane/ethyl acetate 30:1) and PtMe(nC8F17)(Et-COD)t (199 mg, 260 µmol) was obtained as a beige solid in 47% yield. R_f = 0.35 (cyclohexane/ethyl acetate 5:1).

^{1}H NMR (400 MHz, $CDCl_3$, ppm) δ = 5.42–5.22 (m, 2H, C*H*), 4.95 (dd d, ^2JPtH = 43 Hz, ^3JHH = 6.1 Hz, ^4JHH = 2.2 Hz, 1H, C*H*), 2.72–2.49 (m, 4H, CH_2), 2.40–2.11 (m, 5H, CH_2), 1.97–1.91 (m, 1H, CH_2), 1.16 (t, ^3JHH = 7.0 Hz, 3H, CH_3), 0.87 (s d, ^2JPtH = 79 Hz, 3H, PtCH_3); 13C NMR (100 MHz, CDCl3, ppm) δ = 131.0 (*C*quart), 105.7 (–, *C*H major), 105.6 (–, *C*H minor), 103.2 (–, *C*H minor), 103.0 (–, *C*H major), 101.9 (–, *C*H major), 101.8 (–, *C*H minor), 33.8 (–, *C*H2 major), 33.7 (–, *C*H2 minor), 33.0 (–, *C*H2 minor), 32.6 (–, *C*H2 major), 31.3 (–, *C*H2 minor), 31.2 (–, *C*H2,major), 30.5 (–, *C*H2,major), 30.4 (–, *C*H2,minor), 27.0 (–, *C*H2, minor), 26.9 (–, *C*H2, major), 14.0 (+, *C*H3, minor), 12.7 (+, *C*H3, major), 6.6 (+, Pt*C*H3, major); 195Pt NMR (129 MHz, CDCl3, ppm) δ = –3766 (s); 19F NMR (400 MHz, CDCl3, ppm) δ = –126.1-126.0 (m, 2F, CF_2), –122.7–121.2 (m, 8F, CF_2), –115.2–112.7 (m, 2F, CF_2), –95.1–90.8 (m, 2F, CF_2), –80.8–80.7 (m, 3F, CF_3); MS (70 eV, EI) *m/z* (%) = 765 (1) [M+], 748/749/750 (8/10/8) [(M-CH3)+], 327/328/329 (34/24/100) [(M–C8F17-CH3)+]; HRMS ($C_{19}H_{19}F_{17}Pt$): Calcd 765.0863; Found 765.0866; IR (ATR): $\tilde{\nu}$ = 2961 (w), 2893 (vw), 2843 (vw), 1489 (vw), 1436 (vw), 1367 (w), 1321 (vw), 1238 (vw), 1201 (m), 1145 (vs), 1106 (w), 999 (w), 940 (vw), 917 (w), 872 (w), 852 (vw), 821 (vw), 795 (vw), 732 (w), 697 (vw), 636 (m), 550 (m), 527 (w), 454 (vw) cm^{-1}.

Link to selected data that was repeated to approve the identity of the compounds:
https://dx.doi.org/10.14272/reaction/SA-FUHFF-UHFFFADPSC-ARCQLLPCVY-UHFFFADPSC-NUHFF-NLLUO-NUHFF-ZZZ
https://dx.doi.org/10.14272/ARCQLLPCVYIECO-RXRNHWPWSA-N.1

Analysis and References for Compounds Pt10-Pt14

The obtained characterization of the tested compounds Pt10-Pt14 are described below. The compounds were used from a library that was hosted for several years at the Molecule Archive at KIT therefore, selected analysis was repeated to approve the identity of the samples that were used in this study. The data obtained can be accessed through the given DOIs, linking to the gained datasets.

Platinum, dichloro[(1,2,5,6-η)-1,5-cyclooctadien-1-ylphenylmethanone] (Pt10)[v]

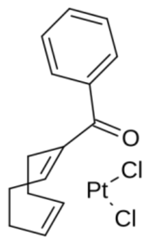

Smiles: O=C(c1ccccc1)C1=CCCC=CCC1.Cl[Pt]Cl; InChIKey: GQRCWUPTTKUWFI-OLPPRYEBSA-L
CAS: 2197920-19-5

^{1}H NMR (400 MHz, $CDCl_3$, ppm): δ = 8.15–8.09 (m, 2H, CH_{Ar}), 7.54 (t, ^{3}J = 7.4 Hz, 1H, CH_{Ar}), 7.46 (t, ^{3}J = 7.5 Hz, 2H, CH_{Ar}), 6.25 (d, ^{3}J = 6.7 Hz, 1H, CH_{COD}), 6.05 (t, ^{3}J = 7.4 Hz, 1H, CH_{COD}), 5.75 (q, ^{3}J = 7.5 Hz, 1H, CH_{COD}), 3.13–3.01 (m, 2H, CH_2), 2.60–2.42 (m, 4H, 2 × CH_2), 2.11–1.97 (m, 2H, CH_2). ^{13}C NMR (100 MHz, $CDCl_3$, ppm): δ = 192.7 (C_q), 139.2 (C_q), 139.1 (C_q), 133.2 (+, 2 × CH_{Ar}), 130.3 (+, CH_{Ar}), 128.7 (+, 2 × CH_{Ar}), 106.1 (+, CH), 103.8 (+, CH), 78.1 (+, CH), 33.9 (−, CH_2), 31.8 (−, CH_2), 29.7 (−, CH_2), 27.8 (−, CH_2). ^{195}Pt NMR (129 MHz, $CDCl_3$, ppm): δ = −3211. MS (70 eV, EI), m/z (%): 442/441 (35/30) [M+], 1874 (35), 105 (100) [$C_7H_5O^+$]. HRMS ($C_{15}H_{16}O_{35}Cl^{195}Pt$): Calcd 442.0532; Found 442.0532. IR (ATR): ṽ = 2961 (w), 1674 (m), 1559 (vw), 1467 (w), 1445 (vw), 1423 (w), 1339 (w), 1312 (vw), 1258 (m), 1229 (w), 1174 (w), 1014 (m), 960 (m), 796 (s), 707 (m), 692 (m), 670 (m), 645 (w), 553 (w), 503 (w), 470 (w), 394 (w) cm^{-1}.

Link to selected data that was repeated to approve the identity of the compounds:
https://dx.doi.org/10.14272/GQRCWUPTTKUWFI-OLPPRYEBSA-L.1

[(1,2,5,6-η)-1-Butyl-1,5-cyclooctadiene]dimethylplatinum (Pt11)[1,vi]

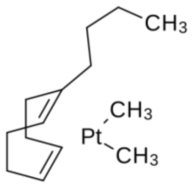

Smiles: CCCCC1=CCCC=CCC1.C[Pt]C; InChIKey: UQRMLGFHZQTIQV-UZUPTRJJSA-N
CAS: 1572182-17-2

^{1}H NMR (400 MHz, CDCl3, ppm): δ = 4.64–4.80 (m, 3H, CH), 2.29–2.52 (m, 4H, CH_2), 2.20–2.26 (m, 2H, CH_2), 2.03–2.18 (m, 3H, CH_2), 1.84–1.99 (m, 1H, CH_2), 1.56–1.62 (m, 1H, CH_2), 1.19–1.38 (m, 3H, CH_2), 0.89 (t, $^{3}J_{HH}$ = 7.2 Hz, 3H, CH_3), 0.69 (s d, $^{2}J_{PtH}$ = 81.5 Hz, 6H, CH_3). ^{13}C NMR (100 MHz, $CDCl_3$, ppm): δ = 120.0 (C_{quart}), 99.3 (+, s d, $^{1}J_{PtC}$ = 46.1 Hz, CH), 97.8 (+, s d, $^{1}J_{PtC}$ = 61.5 Hz, CH), 97.5 (+, s d, $^{1}J_{PtC}$ = 58.3 Hz, CH), 40.2 (–, CH_2), 33.1 (–, CH_2), 31.5 (–, CH_2), 31.0 (–, CH_2), 30.9 (–, CH_2), 28.3 (–, CH_2), 22.7 (–, CH_2), 14.0 (+, CH_3CH_2), 9.5 (+, $PtCH_3$), 3.5 (+, $PtCH_3$). MS (70 eV, EI), m/z (%): 390/389/380 (3/2/2) [M+], 375/374/373 (14/16/14) [M+–CH_3], 359/358/357/356/355/354/353 (32/70/66/85/100/48) [M+– 2 × CH_3]. HRMS ($PtC_{14}H_{26}$): Calcd 389.1682; Found 389.1681. IR (ATR) = 3442 (vs), 2925 (vw), 2873 (s), 2834 (vs), 2797 (vs), 1656 (vs), 1525 (vs), 1480 (vs), 1464 (vs), 1431 (s), 1378 (vs), 1340 (vs), 1315 (vs), 1216 (vs), 1195 (vs), 1167 (vs), 1104 (vs), 1103 (vs), 999 (vs), 929 (vs), 88 (vs), 790 (vs), 730 (vs), 559 (vs), 539 (s) cm^{-1}.

Link to selected data that was repeated to approve the identity of the compounds:
https://dx.doi.org/10.14272/UQRMLGFHZQTIQV-UZUPTRJJSA-N.1

[(1,2,5,6-η)-((2-trimethylsilyl)ethynyl)phenyl-1,5-cyclooctadiene] dimethylplatinum (Pt12)

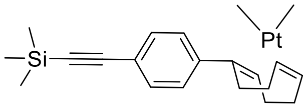

Smiles: C1C=CCCC(=CC1)c1ccc(cc1)C1=CCCC=CCC1.C[Pt]C
InChIKey: HQTKSIZQLFXXCU-GMDPSNEVSA-N
CAS: -

^{1}H NMR (400 MHz, $CDCl_3$, ppm): δ = 7.36 (d, ^{3}J = 8.5 Hz, 2H, CH_{Ar}), 7.29 (d, ^{3}J = 8.5 Hz, 2H, CH_{Ar}), 5.42–5.24 (m, 1H, CH_{COD}), 5.09–5.02 (m, 1H, CH_{COD}), 4.82 (q, ^{3}J = 7.8 Hz, 1H, CH_{COD}), 2.89–2.13 (m, 8H, CH_2), 0.87–0.62 (m, 3H, $PtCH_3$), 0.24 (s, 9H, $SiC(CH_3)_3$), 0.15–0.07 (m, 3H, $PtCH_3$). ^{13}C NMR (100 MHz, $CDCl_3$, ppm): δ = 144.9 (C_q), 131.5 (+, 2 × CH_{Ar}), 126.7 (+, 2 × CH_{Ar}), 121.4 (C_q), 112.0 (C_q), 105.2 (C_q), 99.5 (+, CH), 99.4 (+, CH), 94.8 (+, CH), 94.5 (C_q), 35.6 (−, CH_2), 33.0 (−, CH_2), 32.4 (−, CH_2), 28.1 (−, CH_2), 10.9 (+, $PtCH_3$), 3.8 (+, $PtCH_3$), −0.03 (+, 3 *x* $SiC(CH_3)_3$). MS (70 eV, EI), m/z (%): 505 (21) [M+], 477/476/475/474/473 (24/26//86/100/78), 380 (24), 211 (28). HRMS ($C_{21}H_{30}{}^{195}Pt_2{}^{8}Si$): Calcd 505.1765; Found 505.1766. IR (ATR): ṽ = 2922 (w), 2871 (w), 2154 (w), 1496 (w), 1428 (vw), 1248 (w), 998 (vw), 836 (m), 758 (m), 648 (w), 626 (w), 603 (vw), 561 (w), 539 (w), 417 (vw) cm^{-1}.

Link to selected data that was repeated to approve the identity of the compounds:
https://dx.doi.org/10.14272/IIUHRCTVDJSFPI-STJSRQNLSA-N.1

Bis(dimethyl-η4)-di((1Z,5Z)-cycloocta-1,5-dien-1-yl)-1,1'-biphenyl)diplatinum(II) (Pt13)

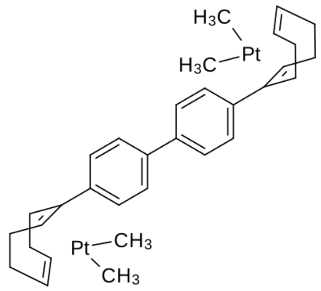

Smiles: C1C=CCCC(=CC1)c1ccc(cc1)c1ccc(cc1)C1=CCCC=CCC1.C[Pt]C.C[Pt]C
InChIKey: IHSHARCKRGLPFN-JSRMEBEPSA-N
CAS: -

^{1}H NMR (400 MHz, $CDCl_3$, ppm): δ = 7.51 (d, ^{3}J = 8.4 Hz, 4H, CH_{Ar}), 7.42 (d, ^{3}J = 8.1 Hz, 4H, CH_{Ar}), 5.47–5.32 (m, 2H, 2 × CH_{COD}), 5.06 (t, ^{3}J = 7.8 Hz, 2H, 2 × CH_{COD}), 4.83 (q, ^{3}J = 7.6 Hz, 2H, 2 × CH_{COD}), 2.93–2.16 (m, 16H, CH_2), 0.76 (s, 6H, 2 × $PtCH_3$), 0.11 (s, 6H, 2 × $PtCH_3$). ^{13}C NMR (100 MHz, $CDCl_3$, ppm): δ = 143.5 (2 × C_q), 139.0 (2 × C_q), 127.2 (+, 4 × CH_{Ar}), 126.2 (+, 4 × CH_{Ar}), 113.1 (2 × C_q), 99.2 (+, 2 × CH), 99.2 (+, 2 × CH), 94.6 (+, 2 × CH), 35.8 (−, 2 × CH_2), 33.0 (−, 2 × CH_2), 32.3 (−, 2 × CH_2), 28.2 (−, 2 × CH_2), 10.9 (+, 2 × $PtCH_3$), 3.4 (+, 2 × $PtCH_3$). MS (70 eV, EI), m/z (%): 816 (3) [M+], 307 (38), 289 (22), 155/154 (31/100), 136 (61). HRMS ($C_{32}H_{42}{}^{195}Pt_2$): Calcd 816.2582; Found 816.2585. IR (ATR): ṽ = 2920 (w), 2868 (w), 1686 (vw), 1523 (vw), 1487 (vw), 1429 (vw), 1340 (vw), 1314 (vw), 1195 (vw), 1093 (vw), 1034 (vw), 1002 (vw), 931 (vw), 867 (vw), 849 (vw), 824 (w), 805 (w), 780 (w), 725 (vw), 609 (vw), 562 (w), 540 (w), 521 (w), 473 (vw), 405 (vw) cm^{-1}.

Link to selected data that was repeated to approve the identity of the compounds:
https://dx.doi.org/10.14272/IHSHARCKRGLPFN-JSRMEBEPSA-N.1

Bis(dimethyl-η4)-((1Z,1'E,5Z,5'E)-(1,1'-bi(cyclooctadiene))diplatinum(II) (Pt14)

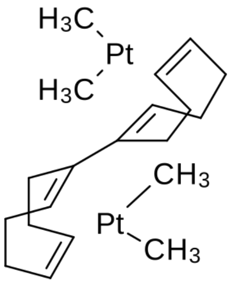

Smiles: C1C=CCCC(=CC1)C1=CCCC=CCC1.C[Pt]C.C[Pt]C
InChIKey: PHIWIZAHOBWKFJ-HPCBIOCFSA-N
CAS: -

^{1}H NMR (400 MHz, $CDCl_3$, ppm): δ = 5.08–4.90 (m, 4H, CH_{COD}), 4.74 (td, ^{3}J = 8.3 Hz, 6.0 Hz, 2H, CH_{COD}), 2.95 (dt, ^{3}J = 14.7 Hz, 5.8 Hz, 2H, CH_2), 2.66–2.40 (m, 8H, CH_2), 2.30–2.04 (m, 6H, CH_2), 0.69 (s, 6H, 2 × $PtCH_3$) 0.53 (s, 6H, 2 × $PtCH_3$). ^{13}C NMR (100 MHz, $CDCl_3$, ppm): δ = 116.6 (2 × C_q), 99.7 (+, 2 × CH), 99.3 (+, 2 × CH), 93.5 (+, 2 × CH), 34.4 (−, 2 × CH_2), 32.8 (−, 2 × CH_2), 31.1 (−, 2 × CH_2), 27.7 (−, 2 × CH_2), 9.0 (+, 2 × $PtCH_3$), 3.9 (+, 2 × $PtCH_3$). MS (70 eV, EI), m/z (%): 665 (23) [M+], 606/605/604/603/602/601 (40/63/57/54/39/46/46), 181 (62), 97 (43), 85 (36), 83 (38), 71 (39), 69 (100), 57 (55). HRMS ($C_{20}H_{34}{}^{195}Pt_2$): Calcd 664.1956; Found 664.1957. IR (ATR): ṽ = 2921 (m), 2871 (w), 1730 (vw), 1522 (vw), 1474 (w), 1428 (w), 1341 (w), 1309 (w), 1291 (vw), 1260 (vw), 1192 (w), 1163 (vw), 1092 (vw), 997 (w), 985 (w), 930 (vw), 865 (w), 804 (w), 788 (w), 769 (w), 731 (w), 603 (vw), 580 (vw), 559 (w), 538 (w), 490 (vw), 444 (vw) cm^{-1}.

Link to selected data that was repeated to approve the identity of the compounds:
https://dx.doi.org/10.14272/PHIWIZAHOBWKFJ-HPCBIOCFSA-N.1

Microbial Strains

ATCC strains were sourced from the American Type Culture Collection and NRS strains from NARSA (Network on Antimicrobial Resistance in Staphylococcus aureus) via BEI Resources (www.beiresources.org), with clinical MSSA and MRSA isolates from Prof Graeme Nimmo, Queensland Health Central Pathology. *Candida albicans* (ATCC 90028, NCCLS 11), *Cryptococcus neoformans* (ATCC 208821, H99) were obtained from the American Type Culture Collection (ATCC). The strains were maintained on glycerol/YPD (Yeast Extract-Peptone Dextrose) broth (20/80 v/v) at -80 °C.

Antibacterial minimum inhibitory concentration (MIC) assay

Compound Assay Plate Preparation: Compounds were dissolved in DMSO at 10mM (CO-ADD screening) or 4 mM (follow up screening) and were plated in a 2-fold dose response from 20 to 0.156 µM or 200 to 0.006 µM respectively, with a maximum of 0.5% or 1% DMSO, final in the assay concentration.

Compound preparation for results in table 3- Compounds were dissolved in DMSO at 4mM and were plated in a 2-fold dose response from 200 to 0.006 µM, with a maximum of 1 % DMSO, final in the assay concentration. (in-house screening)

For all the bacterial assays, each strain was cultured in Cation-adjusted Mueller Hinton broth (CAMHB; Bacto Laboratories 212322) at 37 °C overnight. A sample of each culture was then diluted 40-fold in

fresh CAMHB and incubated at 37 °C for 1.5–3 h. The resultant mid-log phase cultures were diluted with CAMHB (CFU mL^{-1} measured by OD_{600}), then added to each well of the compound-containing plates (384-well non-binding surface (NBS) plates; Corning CLS3640), giving a cell density of 5×10^5 CFU mL^{-1} and a total volume of 50 µL. Plates were covered and incubated at 37 °C for 18 h without shaking. Inhibition of bacterial growth was determined measuring absorbance at 600 nm (OD_{600}), using media only as negative control and bacteria without inhibitors as positive control. MIC values were determined as the lowest concentration at which the growth was inhibited at ≥80%. Colistin sulfate (Sigma C4461) and vancomycin HCl (Sigma 861987) were used as internal controls on each plate for Gram-negative and Gram-positive bacteria, respectively.

Cytotoxicity assay

HEK-293 ATCC CRL-1573 human embryonic kidney cells suspended in DMEM media (Gibco; 11330332) supplemented with 10% FBS (GE; SH30084.03) and 100 U/mL each Penicillin/Streptomycin (Invitrogen; 15070063) were counted manually in a Neubauer haemocytometer and seeded into 384-well, black wall, clear bottom tissue culture plates (Corning; Cat. No. 3712) at 5'000 cells per well in a volume of 20 µL. Manually, 20 µL of each compound dilution was plated in duplicate on the cells, for a final concentration range of 0.8 – 100 µM. The cells were incubated together with the compounds for 20 h at 37 °C, 5% CO_2.

Cytotoxicity (or cell viability) was measured by fluorescence, ex: 560/10 nm, em: 590/10 nm (F560/590), after addition of 5 µL of 25 µg/mL resazurin (2.3 µg/mL final concentration; Sigma R7017) and after further incubation for 3 h at 37 °C in 5% CO_2, using media only as negative control and cells without inhibitors as positive control. CC_{50} (concentration at 50% cytotoxicity) were calculated by curve fitting the inhibition values vs. log(concentration) of four replicates using a sigmoidal dose-response function, with variable fitting values for bottom, top and slope using Prism 8. Tamoxifen (Sigma T5648) was used as internal control on each plate.

Haemolysis assay

Human whole blood (Australian Red Cross Blood Service) was washed three times with 3 volumes of 0.9% NaCl and resuspended in a concentration of 0.5×10^8 cells/mL, determined by manual cell count in a Neubauer haemocytometer. Washed cells were added to compound containing plates (384-well round bottom polypropylene plates, Corning 3657) for a final volume of 50 µL, shaken for 10 min and incubated for 1 h at 37 °C, without shaking. After incubation, the plates were centrifuged at 1000 g for 10 min to pellet cells and debris, 25 µL of the supernatant was then transferred to reading plates (384-well flat bottom polystyrene plates, Corning CLS3680), with haemolysis determined by measuring the supernatant absorbance at 405 mm (OD_{405}), using cells without inhibitors as negative control and cells with 1% Triton X-100 (Sigma T8787) as positive control. HC_{10} and HC_{50} (concentration at which 10% and 50% haemolysis is induced, respectively) were calculated by curve fitting the inhibition values vs. log(concentration) of four replicates using a sigmoidal dose-response function with variable fitting values for top, bottom and slope using Prism 8. Melittin (Sigma M2272) was used as positive haemolytic control on each plate. Human ethics approval from the University of Queensland Institutional Human Research

Ethics Committee was obtained for use of human blood for haemolysis studies (approval number 201400003).

In vivo tox (moth)

G. mellonella larvae were reared in controlled environmental room at Macquarie University, Sydney, Australia at 26 °C and 65% humidity with a 12-hours light/dark cycle. Larvae (~200 mg) were individually injected with 10 µL of chemical into the last right proleg using a 100 µL syringe (Hamilton Ltd). The injection was done for compounds **1-3**. Each compound was dissolved in DMSO and diluted to final concentrations of 1 µM, 100 µM and 10 mM. We injected 5 larvae for each dilution of each compound. Larvae injected with different dilutions of DMSO (10^{-1}, 10^{-3} and 10^{-5}) were included as negative controls. Following injection, the larvae were incubated at 26 °C and monitored every 24 h for 4 days. Larval performance was assessed according to the *G. mellonella* Health Index Scoring System.[1] The experiments were repeated three separate times.

In vivo efficacy (moth)

Galleria larvae (weighing between 200-250 mg) were infected with 10^7 cells of *Staphylococcus aureus* (ATCC 43300, MRSA) (suspended in 10 µL PBS) and incubated at 37 °C. Two hours later, 10 µL compounds of either **Pt1** or **Pt2** (0.4 mM) in 10% DMSO was injected to the other proleg of the larvae. Larvae injected with 10% DMSO and Rifampicin (0.4 mg/mL) were included as controls. The experiment was performed with 15 larvae/ treatment. At 24 h after drug administration, hemolymph of individual larva was collected in PBS and serial dilutions (1:10, 1:100 and 1:1000) were prepared. Bacterial number was determined by drop plate method. Briefly, 5 µL of each dilution was dropped in Mueller-Hinton agar (n=3) and incubated at 37 °C. Bacterial number was counted after incubation for 24 h.

Table S1. Antibacterial activity (MIC ([μM]) of selected complexes against a panel of Gram-positive bacteria.

Species	Strain	Pt3	Pt4	Pt5	Pt6	Pt7	Pt8	Pt9	Pd1	Van[#]
S. aureus[a]	ATCC 25923, MSSA	50	100,00	100,00	100,00	>200	25,00	n.d.	200	1
S. aureus[b]	ATCC 43300, MRSA	0.78-1.56	6,25	25-50.0	6,25	200	3.125	25-50.0	100-200	0.5-1
S. aureus[b]	NRS 17, GISA	3.125-6.25	6.25-12.5	100	25-50.0	200	50	100	100-200.0	8
S. aureus[a]	VRS-1, VRSA (VanA)	>200	>200	200,00	200,00	>200	12,50	n.d.	>200	>64
S. epidermidis[a]	ATCC 14990, type strain	6.25	50,00	50,00	100,00	200,00	6,25	n.d.	100	1-2
S. epidermidis[a]	NRS 60, VISE (VanA)	25	100,00	50,00	200,00	>200	25,00	n.d.	100-200	4
E. faecium[a]	ATCC 35667, type strain	200	>200	>200	>200	>200	100,00	n.d.	>200	0.5
E. faecium[b]	ATCC 51559, MDR, VRE (VanA)	12.5-25	25	200	100-200.0	200	50	100	200	>64
E. faecium[a]	Clinical Isolate, VRE (VanA)	200->200	200,00	200,00	200,00	>200	100,00	n.d.	>200	>64
E. faecalis[a]	ATCC 29212, control strain	200->200	>200	>200	>200	>200	100,00	n.d.	>200	2-4
E. faecalis[a]	Clinical Isolate, VRE (VanB)	200	>200	>200	>200	>200	50-100,00	n.d.	>200	64
B. subtilis[b]	ATCC 6051, type strain	12.5-50	25-100.0	100-200.0	25-100.0	>200	50	100-200.0	>200	0.5

MSSA – methicillin susceptible *S. aureus*; MRSA – methicillin resistant *S. aureus*; MDR – multidrug resistant; GISA – glycopeptide intermediate *S. aureus*; VRSA – glycopeptide resistant *S. aureus*; VISE – vancomycin intermediate *S. epidermidis*; VRE – vancomycin resistant *Enterococcus*; VanA and VanB are vancomycin resistance genes. [#]Vancomycin, MIC given in µg/mL. [a]MIC determined with n=4. [b]MIC determined with n=2.

Table S2. Antibacterial activity (MIC ([μM]) against *E. coli* membrane mutants.

	E. coli *lpxC*	***E. coli*** *TolC*
**Pt1**	100-200	50-200
**Pt2**	100	50-200
**Pt3**	200	>200
**Pt4**	200->200	200->200
**Pt5**	>200	>200
**Pt6**	200->200	>200
**Pt7**	>200	>200
**Pt8**	>200	50-200
**Pt9**	>200	>200
**Pd1**	>200	50-200
**Colistin [ug/mL]**	≤0.03	≤0.03

Table S3. Antibacterial activity (MIC ([μM]) in the presence of human serum (serum reversal)

	B. subtilis ATCC 6051 type strain	***S. aureus*** ATCC 43300 MRSA	***E. faecium*** ATCC 51559 MDR, VRE	***S. aureus*** NRS 17 GISA
Serum %	10%	50%	10%	50%
**Pt1**	12.5-25	200->200	200->200	>200
**Pt2**	12.5-50	100->200	>200-200	>200
**Pt3**	>200	>200	>200	>200
**Pt4**	>200	>200	>200	>200
**Pt5**	>200	>200	>200	>200
**Pt6**	>200	>200	>200	>200
**Pt7**	>200	>200	>200	>200
**Pt8**	200	>200	>200	>200
**Pt9**	>200	>200	>200	>200
**Pd1**	>200	>200	>200	>200
**Vancomycin [ug/mL]**	0.5	0.5-1	>64	8

References

[1] Enders, Mirja; Goerling, Benjamin; Braun, Alexander B.; Seltenreich, Judith E.; Reichenbach, Linus F.; Rissanen, Kari; Nieger, Martin; Luy, Burkhard; Schepers, Ute; Braese, Stefan, Organometallics (2014), 33(15), 4027-4034. https://doi.org/10.1021/om500540x

[2]Komiya, Sanshiro; Ezumi, Sei; Komine, Nobuyuki; Hirano, Masafumi, Organometallics (2009), 28(13), 3608-3610.

[3] Aggarwal, Vikas; Reichenbach, Linus F.; Enders, Mirja; Muller, Thierry; Wolff, Simone; Crone, Marlene; Tuerk, Michael; Brase, Stefan, From Chemistry - A European Journal (2013), 19(38), 12794-12799.

[4] By Wolff, Simone; Crone, Marlene; Muller, Thierry; Enders, Mirja; Braese, Stefan; Tuerk, Michael, Journal of Supercritical Fluids (2014), 95, 588-596.

[5] Wandler, Angela E. E.; Koos, Martin R. M.; Nieger, Martin; Luy, Burkhard; Braese, Stefan, Dalton Transactions (2018), 47(11), 3689-3692. https://doi.org/10.1039/C8DT00075A

[vi] Matthias Faust Mirja Enders Kun Gao Linus Reichenbach Thierry Muller Wolfgang Gerlinger Bernd Sachweh Gerhard Kasper Michael Bruns Stefan Bräse Martin Seipenbusch, Chem. Vap. Deposition 2013, 19, 274–283. https://doi.org/10.1002/cvde.201207038

[7] a) S. M. Cook, J. D. McArthur, Virulence 2013, 4, 350-353; b) C. J.-Y. Tsai, J. M. S. Loh, T. Proft, Virulence 2016, 7, 214-229.
